# Employing antagonistic C-X-C motif chemokine receptor 4 antagonistic peptide functionalized NaGdF_4_ nanodots for magnetic resonance imaging-guided biotherapy of breast cancer

**DOI:** 10.1038/s41598-024-66645-2

**Published:** 2024-07-09

**Authors:** Xiaodong Li, Yunkai Bao, Zhuheng Li, Peihong Teng, Lina Ma, Hua Zhang, Guifeng Liu, Zhenxin Wang

**Affiliations:** 1 Department of Radiology, China-Japan Union Hospital of Jilin University, 130033 Changchun, People’s Republic of China; 2 State Key Laboratory of Electroanalytical Chemistry, Chinese Academy of Sciences, Changchun Institute of Applied Chemistry, 130022 Changchun, People’s Republic of China; 3 Jilin Provincial Institute of Education, 130024 Changchun, People’s Republic of China; 4 School of Traditional Chinese Medicine, Jilin Agriculture Science and Technology College, 132101 Jilin, People’s Republic of China

**Keywords:** NaGdF_4_ nanodot conjugates, C-X-C motif chemokine receptor 4 antagonistic peptide, T_1_-weighted magnetic resonance imaging, Biotherapy, Medical and clinical diagnostics, Imaging studies

## Abstract

C-X-C motif chemokine receptor 4 (CXCR4) is a promising therapeutic target of breast cancer because it is overexpressed on cell surface of all molecular subtypes of breast cancer including triplenegative breast cancer (TNBC). Herein, CXCR4 antagonistic peptide-NaGdF_4_ nanodot conjugates (termed as anti-CXCR4-NaGdF_4_ NDs) have been constructed for magnetic resonance imaging (MRI)-guided biotherapy of TNBC through conjugation of the C-X-C Motif Chemokine 12 (CXCL12)-derived cyclic peptide with tryptone coated NaGdF_4_ nanodots (5 ± 0.5 nm in diameter, termed as Try-NaGdF_4_ NDs). The as-prepared anti-CXCR4-NaGdF_4_ NDs exhibits high longitudinal relaxivity (*r*_1_) value (21.87 mM^−1^S^−1^), reasonable biocompatibility and good tumor accumulation ability. The features of anti-CXCR4-NaGdF_4_ NDs improve the tumor-MRI sensitivity and facilitate tumor biotherapy after injection in mouse-bearing MDA-MB-231 tumor model in vivo. MRI-guided biotherapy using anti-CXCR4-NaGdF_4_ NDs enables to suppress 46% tumor growth. In addition, about 47% injection dose of anti-CXCR4-NaGdF_4_ NDs is found in the mouse urine at 24 h post-injection. These findings demonstrate that anti-CXCR4-NaGdF_4_ NDs enable to be used as renal clearable nanomedicine for biotherapy and MRI of breast cancer.

## Introduction

Breast cancer is the most common malignancy in women, which arises from uncontrolled proliferation of breast epithelial cells^1^. As a complex and heterogeneous disease, the accurate pathogenesis of breast cancer is still unknown^1^. The clinical treatment options for breast cancer are diversified, including but not limited to surgery, chemotherapy, radiotherapy, hormone therapy and targeted therapy^2–6^. As an aggressive subtype of breast cancers, triple-negative breast cancer (TNBC) are defined by the lack of estrogen receptor (ER) and progesterone receptor (PR) and absence of human epidermal growth factor receptor 2 (HER2) protein overexpression and HER2 gene amplification^7^. Comparison with non-TNBC, TNBC lack all the critical molecular features, and have poor prognosis associated with shorter overall survival of patients^7^. Therefore, it is highly desirable for developing potential methods for diagnosis and treatment of TNBC.

During past three decades, the nanomaterials have been extensively explored in different disciplines including biomedical and bioanalytical fields for generation of theranostics with high efficiency and development of biosensors with excellent analytical performance^8–20^. For instance, one iron oxide nanoparticle (commercial name, Ferumoxytol) has been approved for clinical application by Food and Drug Administration (FDA) USA, and a certain number of nanomedicines (i.e., nanomaterial-based theranostics) are currently under investigation in pre-clinical and/or clinical trial stages^13^. However, there are continuous biosafety concerns on the nanomedicines because many promising nanomedicines are derived from nonbiodegradable inorganic nanoparticles with large size (> 10 nm in diameter), and can retain in body for long time significantly (even more than months) through interactions with mononuclear phagocyte system (MPS) cells (e.g., Kupffer cells) in spleen and liver^21–27^. This drawback can be overcome by the development of nanomedicines with renal clearance pathway since they exhibit chemical medicine-like pharmacokinetic profiles (e.g., totally excreted from body within a relatively short period (several hours to several days)), resulting in minimizing uptaken in MPS cells^11–13,21,22^.

Due to its high soft-tissue contrast, the magnetic resonance imaging (MRI) has been recognized as a powerful non-invasive tool for diagnosis of various diseases including cancers. In order to improve diagnosis sensitivity of MRI, the contrast agents are widely employed to enhance the intensity of MR signal. Gd-chelates are a well-known kind of MRI contrast agents with high T_1_-weighted MRI contrast enhancement capability, and have achieved great clinical and commercial success because trivalent gadolinium ion (Gd^3+^) has seven unpaired electrons with a large magnetic moment^28–31^. Recently, Gd^3+^-contained nanodots (Gd NDs) with small size (< 10 nm in diameter) have emerged as promising competitors to molecular Gd^3+^ compound because Gd NDs can be not only accumulated into tumor through enhanced permeability and retention (EPR) effect, but also excreted from body like Gd-chelates through renal clearance^32–43^. In addition, the Gd NDs can be further functionalized for achieving high tumor-targeting ability and/or MRI-guided therapy of tumor through conjugation of molecules with specific functionalities (e.g., phosphorylated peptides and hydrophilic block copolymer)^41,42^.

C-X-C chemokine receptor 4 (CXCR4) is an important member of CXC chemokine receptor family, which is identified as a cell surface biomarker associated with the multiple malignant tumors, such as breast cancer and glioblastoma^44–48^. Previous studies indicate that antagonists against CXCR4 have shown enormous potential as cancer diagnostic and therapeutic agents^49–53^. For instance, gold nanoparticle (GNPs)-anti-CXCR4 antibody conjugates (termed as cGNPs) can enhance the efficiency of TNBC radiotherapy by increasing oxidative stress and DNA damage because the cellular internalization amount of cGNPs can be increased significantly through the interactions of overexpressed CXCR4 on TNBC cell membrane and conjugated anti-CXCR4 antibodies on GNP surface^52^.

In this study, a CXC ligand 12 (CXCL12)-derived cyclic peptide (sequence motif cyclo[K(1,5-pentanedioic acid)R-(2-Nal)-GY]) functionalized NaGdF_4_ NDs (termed as anti-CXCR4-NaGdF_4_ NDs) were synthesized for MRI-guided biotherapy of tumor through immobilization of the CXCL12-derived cyclic peptide on tryptone coated 5 nm NaGdF_4_ NDs (termed as Try-NaGdF_4_ NDs) via covalent bond. The as-prepared anti-CXCR4-NaGdF_4_ NDs exhibit highly renal-clearable property and low toxicity. Both in vitro experimental results and in vivo small animal experimental results with mouse-bearing MDA-MB-231 tumor indicate that anti-CXCR4-NaGdF_4_ NDs exhibit good tumor-targeted ability and biotherapeutic efficacy of tumor.

## Experimental section

### Reagents and materials

The CXC ligand 12 (CXCL12)-derived cyclic peptide (sequence motif cyclo[K(1,5-pentanedioic acid)R-(2-Nal)-GY], termed as anti-CXCR4 peptide) was purchased from Shanghai Qiangyao biology. The molecular structure of CXC ligand 12 (CXCL12)-derived cyclic peptide was shown in Fig. S1. The details of reagents used were shown in Supporting Information, which were of analytical grade except specific indication. All of reagents were used without further purification. Milli-Q water (18.2 MΩ cm) was used in all experiments.

### Instrumentation

The details of instruments used were shown in Supporting Information.

### Synthesis of anti-CXCR4-NaGdF_4_ NDs

The oleic acid (OA) capped NaGdF_4_ nanodots (termed as OA-NaGdF_4_ NDs) and Try-NaGdF_4_ NDs were synthesized according to previously reported methods with slight modification (see Supporting Information for details)^34,41,54^. For synthesizing anti-CXCR4-NaGdF_4_ NDs, 5 mL Try-NaGdF_4_ NDs solution (1 mg/mL) were mixed with 2.5 mL hydroxy-2,5-dioxopyrrolidine-3-sulfonicacid (sulfo-NHS, 6 mg/mL) and 2.5 mL 1-(3-dimethylaminopropyl)-3-ethylcarbodiimide (EDC, 4 mg/mL) in MES solution (10 mM, pH 5.9) under vigorous stirring, and continually stirred at 25 °C for 1.5 h. 2.5 mL anti-CXCR4peptide solution (1 mg/mL in H_2_O) were then added into the mixture. After vigorously stirred at 25 °C for another 12 h, the mixture was purified by centrifugation (10000 rpm, 10 min, 3 times). The final product (termed as anti-CXCR4-NaGdF_4_ NDs) was redispersed into PBS (10 mM PB containing 137 mM NaCl, pH 7.4). The as-prepared Try-NaGdF_4_ NDs and anti-CXCR4-NaGdF_4_ NDs were characterized by MRI of Phantom and cell viability assay (see Supporting Information for details).

### Cell uptake

The MDA-MB-231 cells (1 × 10^4^ cells per well) were seeded into 96-well plates with 100 µL RPMI 1640 culture medium supplemented with 10% (wt/v) FBS and 100 U/mL chlorostreptomycin. After the cells were cultured for 24 h, the culture medium was replaced by 100 μL of fresh culture medium containing various concentrations of anti-CXCR4-NaGdF_4_ NDs and Try-NaGdF_4_ NDs, respectively. After incubated for another 24 h, the NDs contained culture medium was discharged. The NDs stained cells were washed by 100 µL fresh culture medium (3 times), and 100 µL PBS, detached by 100 μL trypsin, counted by cell counter, and centrifuged at 2000 r/min for 5 min. 1 × 10^6^ NDs stained MDA-MB-231 cells were carefully dispersed in 1% agarose hydrogel for in vitro MRI (see Supporting Information for details).

### In vivo measurements

BALB/c nude mice with average bodyweight of 20 g were purchased from Beijing Vital River Laboratory Animal Technology Co., Ltd., (Beijing, China). All small animal experiments were in agreement with the guidelines of the Regional Ethics Committee for Animal Experiments established by Changchun Institute of Applied Chemistry Institutional Animal Care and Use (Ref. No. 20240007). The MDA-MB-231 tumor models were achieved by inoculating subcutaneously 5 × 10^6^ MDA-MB-231 cells suspended in PBS (100 μL) to the right flanks of BALB/c nude mice.

For in vivo tumor accumulation study, MDA-MB-231 tumor-bearing BALB/c nude mice were anesthetized by using chloral hydrate (10 wt%), pre-injected intravenously with desired amounts (10 mg [Gd] kg^−1^ body weight) of anti-CXCR4-NaGdF_4_ NDs and Try-NaGdF_4_ NDs in PBS (100 μL) through tail vein, respectively, and acquired the T_1_-weighted MR images at desired time points after injection by a Siemens 1.5 T MRI scanner (see Supporting Information for details).

For biodistribution analysis, the MDA-MB-231 tumor-bearing BALB/c nude mice were sacrificed at 2 and 24 h post injection of the anti-CXCR4-NaGdF_4_ NDs and Try-NaGdF_4_ NDs. The tissues (tumor, heart, liver, spleen, lung and kidneys) were collected and digested in aqua regia at 80 °C for 2 h. The amounts of Gd element in the as-obtained liquids were detemined by inductively coupled plasma mass spectrometer (ICP-MS).

For biotherapy, the BALB/c nude mice with tumor sizes of 3–4 mm in diameter were divided into four randomized groups, which were treated by PBS, anti-CXCR4-peptide, Try-NaGdF_4_ NDs and anti-CXCR4-NaGdF_4_ NDs, respectively. The tumor sizes (tumor length and tumor width) were measured by a caliper for 21 days. The tumor volumes were calculated by (tumor length) × (tumor width)^2^/2. The relative tumor volumes were calculated as V/V_0_ (V_0_ was the tumor volume when the treatment was initiated). The tumors were collected, and recorded by a digital camera at 21th day post-injection. In addition, the MDA-MB-231 tumor-bearing mice were treated as descripted above, and the Kaplan–Meier survival curves were recorded within 40 days after treatment for evaluation of survival rate.

For toxicology analysis, healthy mice were treated by the anti-CXCR4-NaGdF_4_ NDs and Try-NaGdF_4_ NDs in PBS (100 μL) at a dose of 10 mg [Gd] kg^−1^ body weight through intravenous administration, respectively. Mice treated with PBS were used as the control group. The mice were sacrificed at 30th day post-injection, and the tissues including heart, spleen, liver, lung and kidneys were fixed in 10% neutral buffered formalin for the histological analysis*.*

## Results and disscussion

### Synthesis and characterization of anti-CXCR4-NaGdF_4_ NDs

**Scheme 1:**
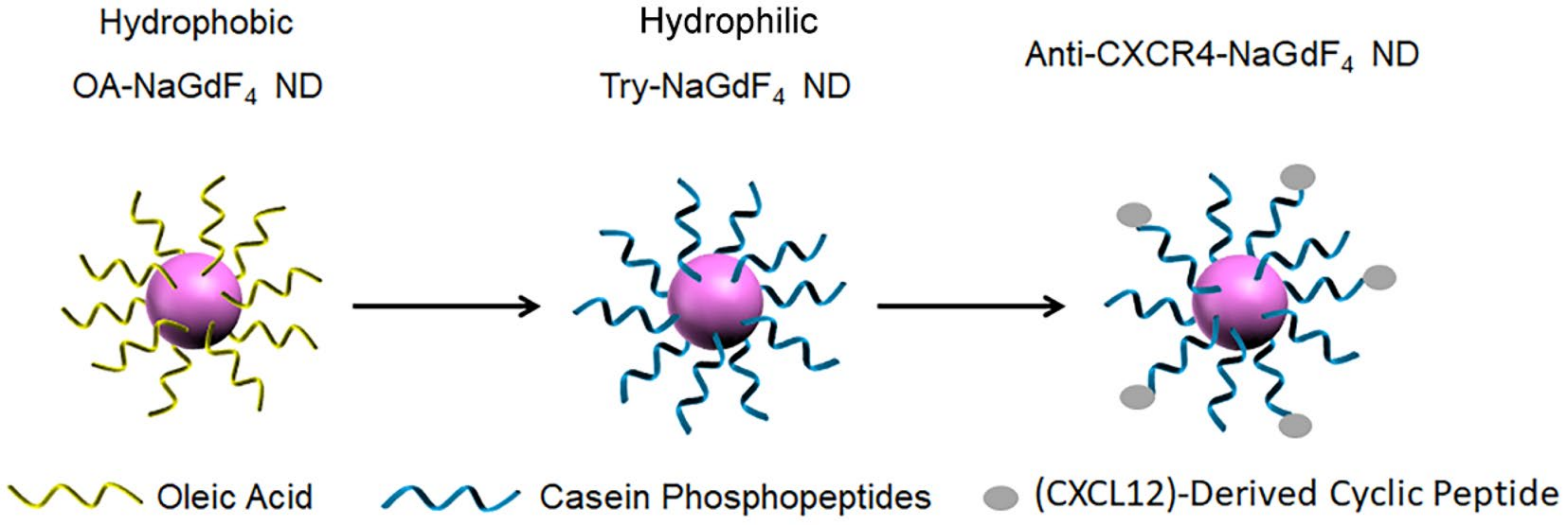
The schematic presentation of the synthsis procedure of anti-CXCR4-NaGdF_4_ NDs.

**Figure 1:**
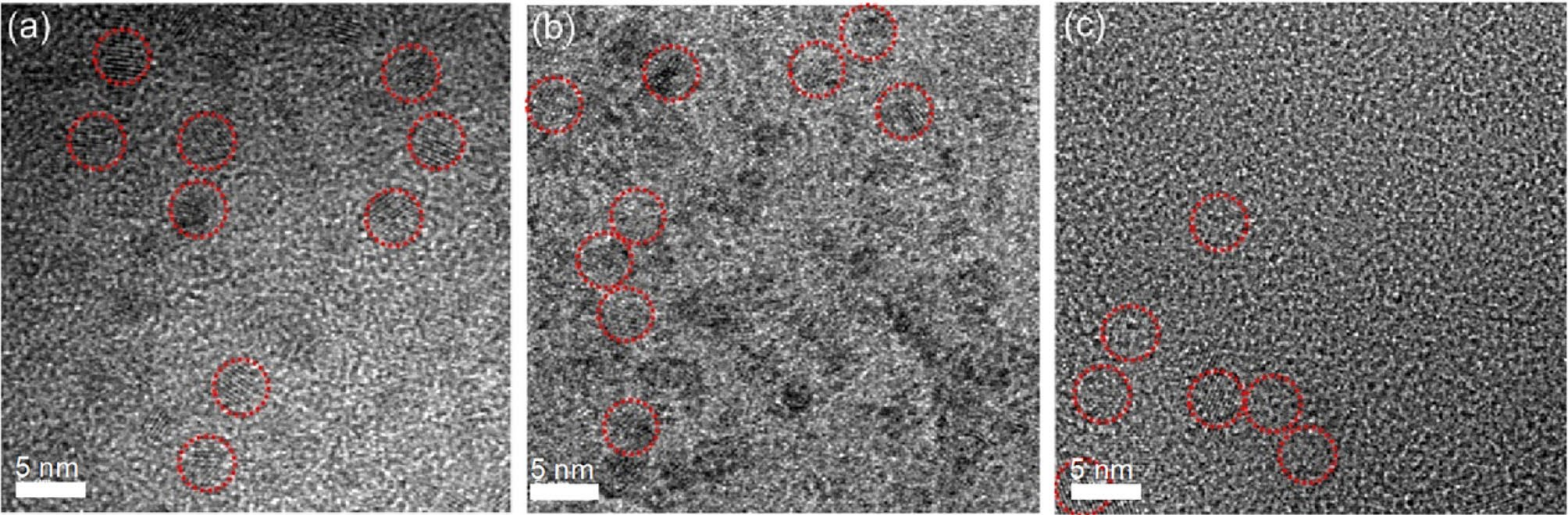
The TEM micrographs of as-synthesized (**a**) OA-NaGdF_4_ NDs, (**b**) Try-NaGdF_4_ NDs and (**c**) anti-CXCR4-NaGdF_4_ NDs.

**Figure 2:**
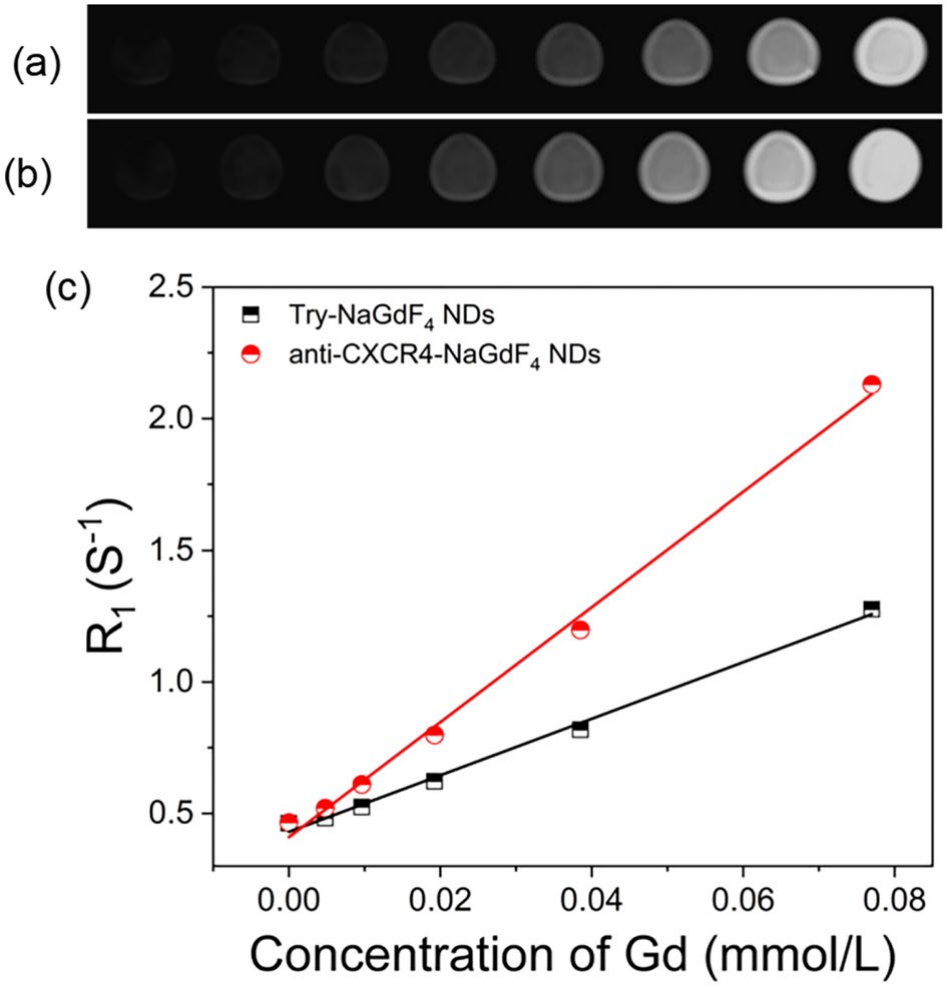
The MR images of (**a**) Try-NaGdF_4_ NDs and (**b**) anti-CXCR4-NaGdF_4_ NDs in solution. (**c**) R_1_ relaxivities of anti-CXCR4-NaGdF_4_ NDs (red line, *r*_1_ = 21.87 mM^−1^ S^−1^) and Try-NaGdF_4_ NDs (black line, *r*_1_ = 10.75 mM^−1^ S^−1^) as a function of the molar concentration of Gd in the solution.

The synthetic procedure and biomedical application of anti-CXCR4-NaGdF_4_ NDs are shown in Scheme 1. Briefly, the hydrophobic OA-NaGdF_4_ NDs were synthesized by reported solvothermal methods^34,41,54^. The hydrophilic Try-NaGdF_4_ NDs were easily prepared through mixing hydrophobic OA-NaGdF_4_ NDs with tryptone at room temperature under vigorous stirring. Since tryptone contains large amounts of the casein phosphopeptides (CPPs) with motif -Ser(P)-Ser(P)-Ser(P)-Glu-Glu-, the hydrophobic OA molecules on the surface of NaGdF_4_ NDs were replaced by the hydrophilic tryptone via formation of robust Gd^3+^-phosphate coordination bonds^34^. Subsequently, the carboxyl groups of tryptone on Try-NaGdF_4_ NDs were activated by EDC and sulfo-NHS. Finally, the anti-CXCR4-NaGdF_4_ NDs were prepared by the amidation reaction between activated carboxyl group of Try-NaGdF_4_ NDs and the amine group of anti-CXCR4 peptide. There are negligible change of the morphology, size and crystalline nature of NaGdF_4_ NDs after ligand exchange and anti-CXCR4 functionality (as shown in Fig. 1). The average size of as-prepared NaGdF_4_ NDs is 5 ± 0.5 nm in diameter. The Zeta potentials of Try-NaGdF_4_ NDs and anti-CXCR4-NaGdF_4_ NDs are −5.1 ± 0.6 mV and 5.8 ± 0.8 mV, respectively (as shown in Fig. S2). The result is consistent with the negatively charged nature of phosphopeptide outlayer of Try-NaGdF_4_ NDs and the positively charged nature of anti-CXCR4 peptide (PI = 10.5) outlayer of anti-CXCR4-NaGdF_4_ NDs. In addition, the longitudinal relaxivity (r_1_) value of anti-CXCR4-NaGdF_4_ NDs (21.87 mM^−1^S^−1^) is much higher than that of Try-NaGdF_4_ NDs (10.75 mM^−1^S^−1^, as shown in Fig. 2). This result indicates that anti-CXCR4 enhances the interactions of H_2_O molecules and NaGdF_4_ NDs, which may be due to the strong hydrogen bonding of polar amino acids (lysine, tyrosine and arginine) in the monocyclic peptide and H_2_O (as shown in Fig. S3, the hydrogen bond energies between the side chain of lysine, tyrosine, arginine and H_2_O molecules were 21.16, 20.65, 22.14 kJ/mol, respectively). The phenomenon suggests that anti-CXCR4-NaGdF_4_ NDs have strong T1-weighted MRI contrast enhancement capability.

### In vitro* studies*

**Figure 3:**
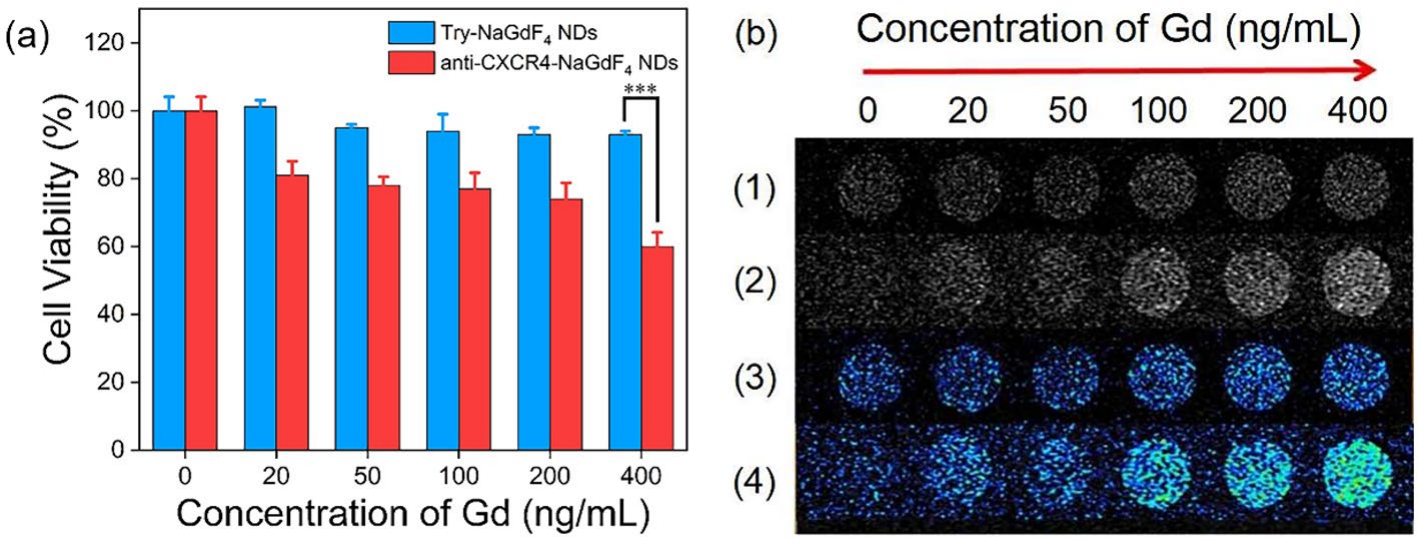
(**a**) In vitro cytotoxicity of Try-NaGdF_4_ NDs and anti-CXCR4-NaGdF_4_ NDs, (**b**) the T_1_-weighted MR images (1, 2) and corresponding pseudo-color images (3, 4) of Try-NaGdF_4_ NDs (1 and 3) and anti-CXCR4-NaGdF_4_ NDs (2 and 4) stained MDA-MB-231 cells (***p < 0.001).

MDA-MB-231 cell line was selected for evaluating the interaction of anti-CXCR4-NaGdF_4_ NDs with cancer cells because the human TNBC is a typical refractory tumor. The MDA-MB-231 cells exhibit less than 70% viability after incubated with 400 ng mL^−1^ anti-CXCR4-NaGdF_4_ NDs for 24 h, while the cells exhibit more than 95% viability after incubated with 400 ng mL^−1^ Try-NaGdF_4_ NDs for 24 h (as shown in Fig. 3a). The result indicates that the anti-CXCR4-NaGdF_4_ NDs have relatively high cytotoxicity towards MDA-MB-231 cells, and can be used as biotherapy agent for killing MDA-MB-231 cells. The T_1_-weighted MR signal intensity of anti-CXCR4-NaGdF_4_ NDs stained MDA-MB-231 cells was stronger than that of Try-NaGdF_4_ NDs stained MDA-MB-231 cells (as shown in Fig. 3b). The cytotoxicity and MRI results indicate that anti-CXCR4-NaGdF_4_ NDs could be used as efficient agents for T_1_-weighted MRI-guided biotherapy of cancer.

### In vivo tumor-targeting capability of anti-CXCR4-NaGdF_4_ NDs

**Figure 4:**
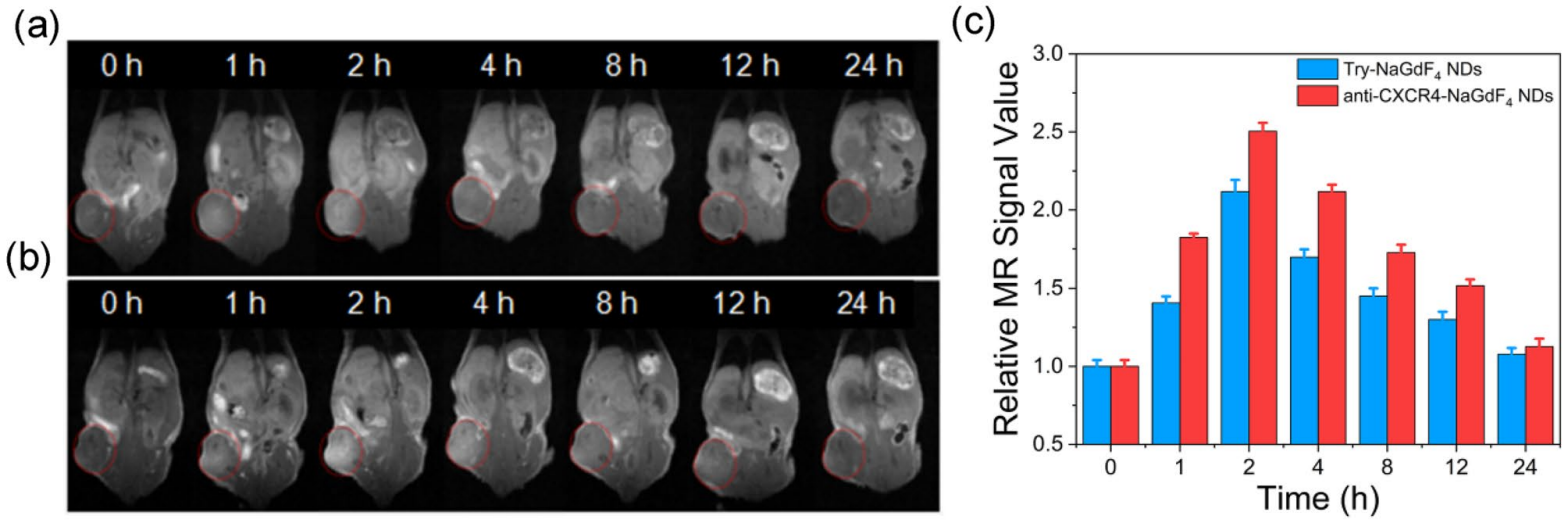
In vivo MR images of BALB/c mice bearing MDA-MB-231 tumor after intravenous injection of (**a**) Try-NaGdF_4_ NDs and (**b**) anti-CXCR4-NaGdF_4_ NDs (10 mg [Gd] kg^−1^ body weight) at preinjection (0), 1, 2, 4, 8, 12 and 24 h post-injection, and (**c**) corresponding data analysis of the MR signal measurements of tumor, respectively. The preinjection (0) signal change is defined as 1.

**Figure 5:**
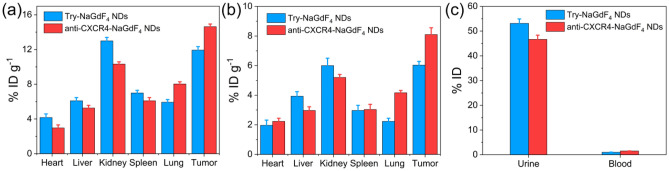
ICP-MS measurements of Gd amounts in BALB/c mice bearing MDA-MB-231 tumor after intravenous injection of Try-NaGdF_4_ NDs and anti-CXCR4-NaGdF_4_ NDs (10 mg [Gd] kg^−1^ body weight) at (**a**) 2 h and (**b**) 24 h post-injection. (**c**) ICP-MS determination of Gd levels in urine and blood of BALB/c mice bearing MDA-MB-231 tumor 24 h after intravenous administration of Try-NaGdF_4_ NDs and anti-CXCR4-NaGdF_4_ NDs (10 mg [Gd] kg^−1^ body weight).

The BALB/c nude mouse bearing MDA-MB-231 tumor was used for evaluating the tumor-targeting capacity of anti-CXCR4-NaGdF_4_ NDs through intravenous injection of anti-CXCR4-NaGdF_4_ NDs and Try-NaGdF_4_ NDs via tail vein, respectively. The T_1_-weighted MR images were then collected at different timed intervals within 24 h post-injection. After administration of NaGdF_4_ NDs, the T_1_-weighted MR signals of tumor sites were increased by incresasing the time between 0 and 2 h post-injection, and gradually decreased after 2 h post-injection (as shown in Figs. 4 and S4). In particular, the T_1_-weighted MR signal intensities in tumor site of anti-CXCR4-NaGdF_4_ NDs treated mice are stronger than those of Try-NaGdF_4_ NDs treated mice at all timed intervals within 12 h post-injection. The phenomenon may be due to the interaction between anti-CXCR4 peptide and over expressed CXCR4 on MDA-MB-231 cells. The result suggests that anti-CXCR4-NaGdF_4_ NDs can be used as a T_1_-weighted MRI contrast agent with wide imaging window (at least 12 h) for detection of CXCR4 overexpression tumors (e.g., TNBC). Furthermore, anti-CXCR4-NaGdF_4_ NDs and Try-NaGdF_4_ NDs treated tumor-bearing mice were sacrificed at 2 and 24 h post-injection, respectively, and the amounts of Gd in tumors were determined by the ICP-MS measurements. As shown in Fig. 5, the amounts of Gd in tumors of anti-CXCR4-NaGdF_4_ NDs treated mice are higher than those in Try-NaGdF_4_ NDs treated mice. The ICP-MS experimental result confirms the high tumor-targeting capability of anti-CXCR4-NaGdF_4_ NDs in tumor.

In addition, the MR signal intensities in kidneys and liver of Try-NaGdF_4_ NDs and anti-CXCR4-NaGdF_4_ NDs treated mice were also collected (as shown in Figs. S5, S6). The changes of MR signals of kidneys in NDs treated mice are similar to those of tumors in NDs treated mice. The MR signals of liver in NDs treated mice show negligible changes at all timed intervals. The results indicate that both Try-NaGdF_4_ NDs and anti-CXCR4-NaGdF_4_ NDs are excreted by renal clearance. In addition, the MR signal intensities in kidneys of Try-NaGdF_4_ NDs treated mice are stronger than those of anti-CXCR4-NaGdF_4_ NDs treated mice at all timed intervals within 24 h post-injection, indicating that the clearance rate of Try-NaGdF_4_ NDs is faster than that of anti-CXCR4-NaGdF_4_ NDs. This phenomenon may be caused by the interaction among of anti-CXCR4 peptide and overexpressed CXCR4 in tumor.

### In vivo biodistribution and biotoxicity of anti-CXCR4-NaGdF_4_ NDs

For further studying their biodistribution and clearance pathway, the Try-NaGdF_4_ NDs and anti-CXCR4-NaGdF_4_ NDs treated mice were sacrificed at 2 and 24 h post-injection, respectively. The amounts of Gd element in tumors, main organs, blood and urine were measured by ICP-MS. As shown in Fig. 5, the amounts of Gd in kidneys are higher than those in other organs during the whole period. More than 47% ID of NDs were found in the urine at 24 h post-injection. In addition, the amounts of Gd in blood of anti-CXCR4-NaGdF_4_ NDs treated mice are higher than those in Try-NaGdF_4_ NDs treated mice. The result is consistent with that of MRI experiment, which confirms the renal clearance of Try-NaGdF_4_ NDs and anti-CXCR4-NaGdF_4_ NDs. The efficient renal clearance of anti-CXCR4-NaGdF_4_ NDs enables to eliminate potential long-term in vivo toxicity. Furthermore, the healthy BALB/c mice were treated with a single dose of anti-CXCR4-NaGdF_4_ NDs, and sacrificed for histology analysis at 30-day post-injection. In comparison with the control group, negligible lesions and/or abnormalities were observed, indicating low in vivo toxicity of anti-CXCR4-NaGdF_4_ NDs (as shown in Fig. S7).

### Biotherapy of anti-CXCR4-NaGdF_4_ NDs

**Figure 6:**
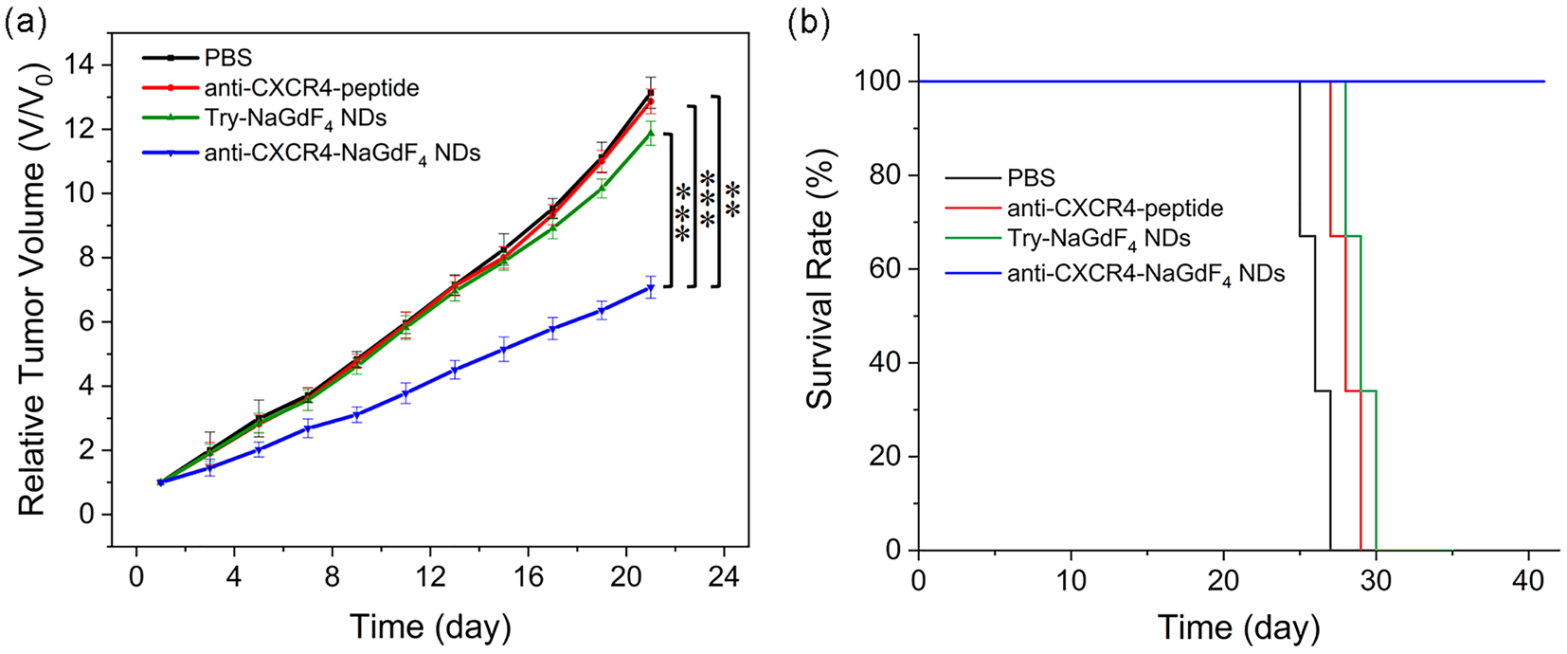
(**a**) The tumor growth curves of mice under different treatments (**p < 0.01, ***p < 0.001). (**b**) The survive curves of MDA-MB-231 tumor-bearing mice after treated by PBS only, anti-CXCR4-peptide, Try-NaGdF_4_ NDs and anti-CXCR4-NaGdF_4_ NDs, respectively. The injection dose of anti-CXCR4-peptide is 1 mg/mL. The injection dose of NDs is 10 mg [Gd] kg^−1^ body weight.

The MDA-MB-231 tumor-bearing mice were randomly divided into four groups, which were treated by PBS only, anti-CXCR4-peptide, Try-NaGdF_4_ NDs and anti-CXCR4-NaGdF_4_ NDs, respectively. After treatments, the tumor volumes were measured every 2 days for 21 days. As shown in Figs. 6 and S8, anti-CXCR4-NaGdF_4_ NDs reveal partial tumor growth inhibition. The survival rates of MDA-MB-231 tumor-bearing mice are strongly dependent on the treatment modes. In particular, the 40-day survival rate of anti-CXCR4-NaGdF_4_ NDs treated reached 100%, which is much higher than those of other groups (PBS only, anti-CXCR4-peptide and Try-NaGdF_4_ NDs). The results indicate that the anti-CXCR4-NaGdF_4_ NDs have certain biotherapeutic effects, which could significantly prolong the survival time of MDA-MB-231 tumor-bearing mice.

## Conclusion

In summary, bifunctional anti-CXCR4-NaGdF_4_ NDs have been successfully fabricated for MRI-guided biotherapy of cancer by a two-step procedure through ligand exchange reaction between oleate and CPPs in tryptone, and the amidation reaction between activated carboxyl group of tryptone and amine group of anti-CXCR4. Both in vitro and in vivo experimental results indicate that the as-prepared anti-CXCR4-NaGdF_4_ NDs exhibit high MRI enhancing performance, good MDA-MB-231 tumor suppression capacity and low toxicity in vivo. Furthermore, the anti-CXCR4-NaGdF_4_ NDs can be efficiently excreted from body through renal clearance. Therefore, the anti-CXCR4-NaGdF_4_ NDs could be employed as a targeting nanomedicine for detection and biotherapy of CXCR4-overexpression tumor. However, the biotherapeutic mechanism of anti-CXCR4-NaGdF_4_ NDs has not been clearly addressed, which should be clarified in future.

### Supplementary Information

Supplementary Information.

## Data Availability

The datasets used during the current study are available from the corresponding author on reasonable request.
