# Supplementary material for: Employing antagonistic C-X-C motif chemokine receptor 4 antagonistic peptide functionalized NaGdF4 nanodots for magnetic resonance imaging-guided biotherapy of breast cancer
Source: Sci Rep. 2024 Jul 9;14:15764. doi: 10.1038/s41598-024-66645-2 (PMC11233619; doi:10.1038/s41598-024-66645-2)
Supplement: Supplementary file 1 — Supplementary Information. [file 41598_2024_66645_MOESM1_ESM.docx]

Supporting Information for,

Employing antagonistic C-X-C motif chemokine receptor 4 antagonistic peptide functionalized NaGdF_4_ nanodots for magnetic resonance imaging-guided biotherapy of breast cancer

Xiaodong Li,^1^ Yunkai Bao,^2^ Zhuheng Li,*^3^ Peihong Teng,^1^ Lina Ma,^4^ Hua Zhang,^2^ Guifeng Liu,*^1^ Zhenxin Wang*^2^

^1^Department of Radiology, China-Japan Union Hospital of Jilin University, Changchun 130033, P. R. China;

^2^State Key Laboratory of Electroanalytical Chemistry, Changchun Institute of Applied Chemistry, Chinese Academy of Sciences, Changchun 130022, P. R. China;

^3^Jilin Provincial Institute of Education, Changchun 130024, P. R. China.

^4^School of Traditional Chinese Medicine, Jilin Agriculture Science and Technology College, Jilin 132101, P. R. China.

Contents

1 Additional Experimental Section

2 Additional Figures S1-S8

3 Additional References

1 Additional Experimental Section

*Reagents and Materials.* Sodium oleate was purchased from TCI Ltd. (Japan). Gd_2_O_3_ (99.99%) was obtained from Alfa Aesar. (Ward Hill, USA). Tryptone, 1-octadecene (ODE, 90%), oleic acid (OA, 90%) and RPMI 1640 culture medium were obtained from Sigma-Aldrich Co. (St Louis, USA). Fetal bovine serum (FBS) was obtained from Gibco. (New York, USA). 3-(4,5-dimethylthiazol-2-yl)-2,5-diphenyltetrazolium bromide (MTT) was received from Beijing Dingguo Biotechnology Ltd. (Beijing, China). Other reagents (analytical grade) were purchased from Beijing Chemical Reagents Company (Beijing, China). All reagents were used without further purification. Milli-Q water (18.2 MΩ cm) was used in all experiments.

*Characterization.* Transmission electron microscope (TEM) micrographs and selected area electron diffraction (SAED) patterns were recorded by TECNAI G2 high-resolution transmission electron microscope (FEI Co., USA). Dynamic light scattering (DLS) and Zeta Potential of the as-prepared samples were carried out on a Zetasizer Nano ZS (Malvern Instruments Ltd., UK). The Gd element analysis was conducted with an ELAN 9000/DRC Inductively Coupled Plasma Mass Spectrometer (ICP-MS) (Perkin Elmer, USA). The relaxation times of the samples were carried out on a 9.4 T Bruker avance III 400 MHz nuclear magnetic resonance spectrometer (Bruker Co., Germany).

*Synthesis of* *OA-NaGdF_4_ NDs.* The OA capped NaGdF_4_ nanodots (termed as, OA-NaGdF_4_ NDs) were synthesized according to a previously reported method with slight modification^1-3^. Briefly, the Gd_2_O_3_ was firstly reacted with excess hydrochloric acid (50% v/v) to form GdCl_3_. After the solvent was evaporated completely, the resulting powder was redispersed in H_2_O to yield the GdCl_3_ aqueous stocking solution (1.5 M). 1 mL GdCl_3_ (1.5 M) aqueous solution were injected in a 100 mL flask, and dried by heating. Then, 6 mL OA and 22.5 mL ODE were added into the flask, respectively. The mixture was heated to 140 °C under argon atmosphere to obtain a homogeneous solution. After cooling to 50 °C, 15 mL methanol solution containing NH_4_F (6 mmol) and NaOH (3.75 mmol) was slowly added dropwise into the solution and stirred overnight. And then the methanol was evaporated at 80 °C, and the solution was degassed for 10 min under argon protection. Subsequently, the temperature of the mixture was increased to 250 °C at a rate of 10 °C min^-1^, and maintained at 250 °C for 10 min under argon protection. After cooling to room temperature, the as-prepared OA-NaGdF_4_ NDs were collected by centrifugation (10000 rpm for 10 min), and washed with 10 mL ethanol (three times) by centrifugation (10000 rpm for 10 min). Finally, the OA-NaGdF_4_ NDs were redispersed in cyclohexane or chloroform.

*Synthesis of Try-NaGdF_4_ NDs.* In a typical process, hydrophobic OA-NaGdF_4_ NDs (10 mg in 12 mL cyclohexane) were added into 32 mL H_2_O solution containing 64 mg tryptone, and the mixture was stirred vigorously at 25 °C for 12 h. Afterward, the OA-NaGdF_4_ NDs can be clearly transferred from the cyclohexane layer into the H_2_O layer due to the ligand exchange of OA by tryptone. The as-obtained aqueous phase was transferred into a dialysis bag with a molecular weight cut-off of 8000, and dialyzed in 10 L H_2_O for 72 h. The H_2_O was changed every 8 h in the dialysis process. Subsequently, the dialyzed product was further purified by centrifugation (13000 rpm, 10 min, 3 times, 10 mL H_2_O). The final product (termed as Try-NaGdF_4_ NDs) was redispersed in 10 mL H_2_O.

*MRI of Phantom.* Anti-CXCR4-NaGdF_4_ NDs and Try-NaGdF_4_ NDs aqueous solution with various concentrations were dispersed in 1.5 mL centrifuge tubes, respectively. T_1_-weighted MR images were obtained using clinical GE Signa 1.5-*T* MR unit (General Electric, Milwaukee, WI) with following imaging parameters: 1.2 mm slice thickness, 15 ms echo time (TE), 358 ms repetition time (TR) and 50 mm×50 mm field of view, respectively. In the MR measurement, blank PBS (10 mM PB containing 137 mM NaCl, pH 7.4) was set as control sample.

*Cell viability assay.* The cytotoxicities of anti-CXCR4-NaGdF_4_ NDs and Try-NaGdF_4_ NDs against MDA-MB-231 cells were evaluated by conventional MTT assay. Briefly, the cells were seeded at a density of 1×10^4^ cells per well into 96-well plates with 100 µL RPMI 1640 culture medium supplemented with 10% (wt/v) FBS and 100 U/mL chlorostreptomycin, and cultured for 24 h. After discharged the culture medium, 100 μL of fresh culture medium containing various concentrations of anti-CXCR4-NaGdF_4_ NDs and Try-NaGdF_4_ NDs were added into the wells, respectively, incubated for another 24 h, and discharged the culture medium. The cells were washed by fresh culture medium (100 µL, 3 times). The traditional MTT toxicology assay was used to evaluate the cell viabilities. The cells cultured without NDs were used as control samples.

*In vitro MRI*. 1×10^6^ NDs stained MDA-MB-231 cells were carefully dispersed in 1% agarose hydrogel. MRI were performed using a Siemens 1.5 T MRI scanner (Magnetom Avanto, Siemens, Erlangen, Germany) by a Siemens 1.5 T MRI scanner with imaging parameters: 1.2 mm slice thickness, 15 ms echo time (TE), 358 ms repetition time (TR) and 50 mm×50 mm field of view.

*In vivo measurements.* For in vivo tumour accumulation study, T_1_-weighted MR images of anti-CXCR4-NaGdF_4_ NDs and Try-NaGdF_4_ NDs treated mice were acquired at desired time points after injection using a Siemens 1.5 T MRI scanner (Magnetom Avanto, Siemens, Erlangen, Germany). Imaging parameters were as follows: TR, 358 ms; TE, 15 ms; field of view, 120 mm × 72 mm and slice thickness, 2.0 mm, respectively.

**2 Additional Figures S1-S8**


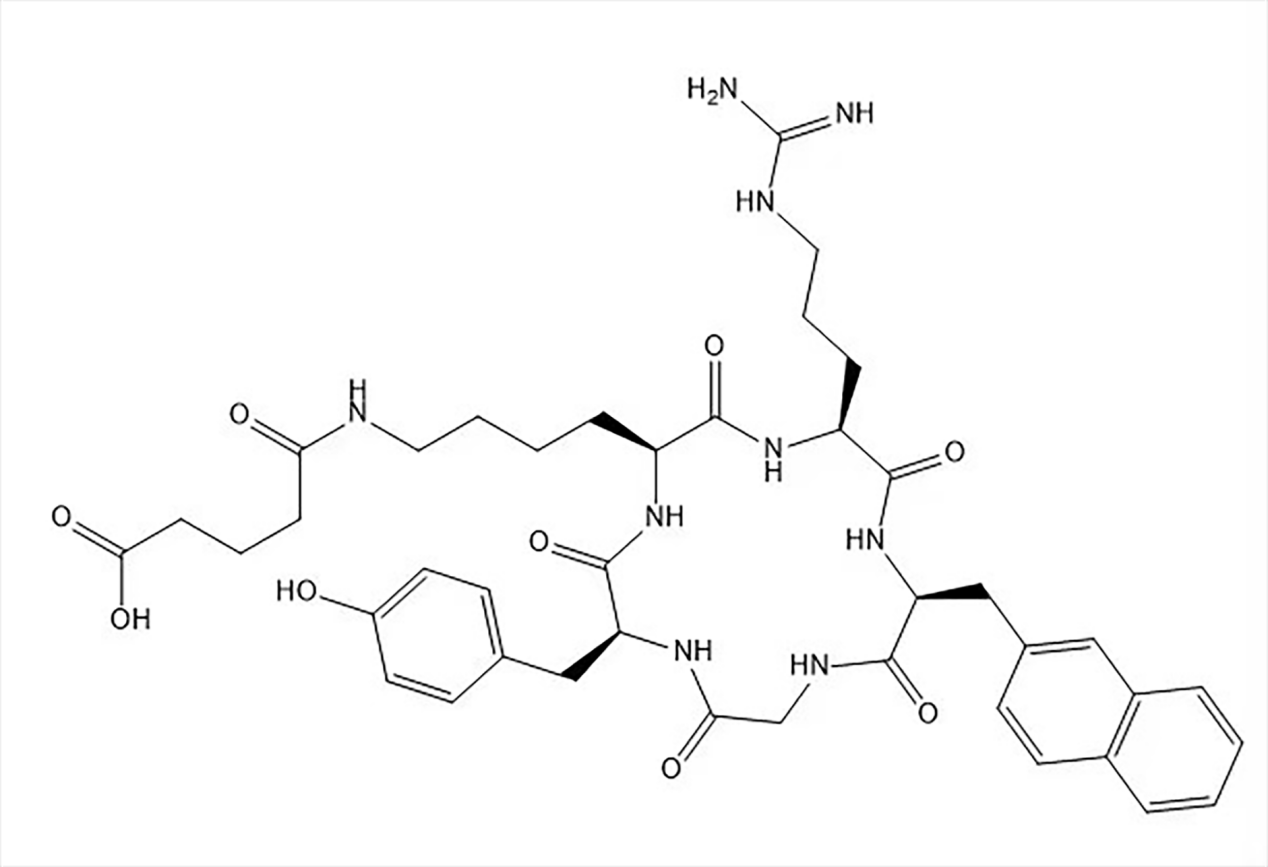


**Figure S1** The molecular structure of anti-CXCR4 peptide.


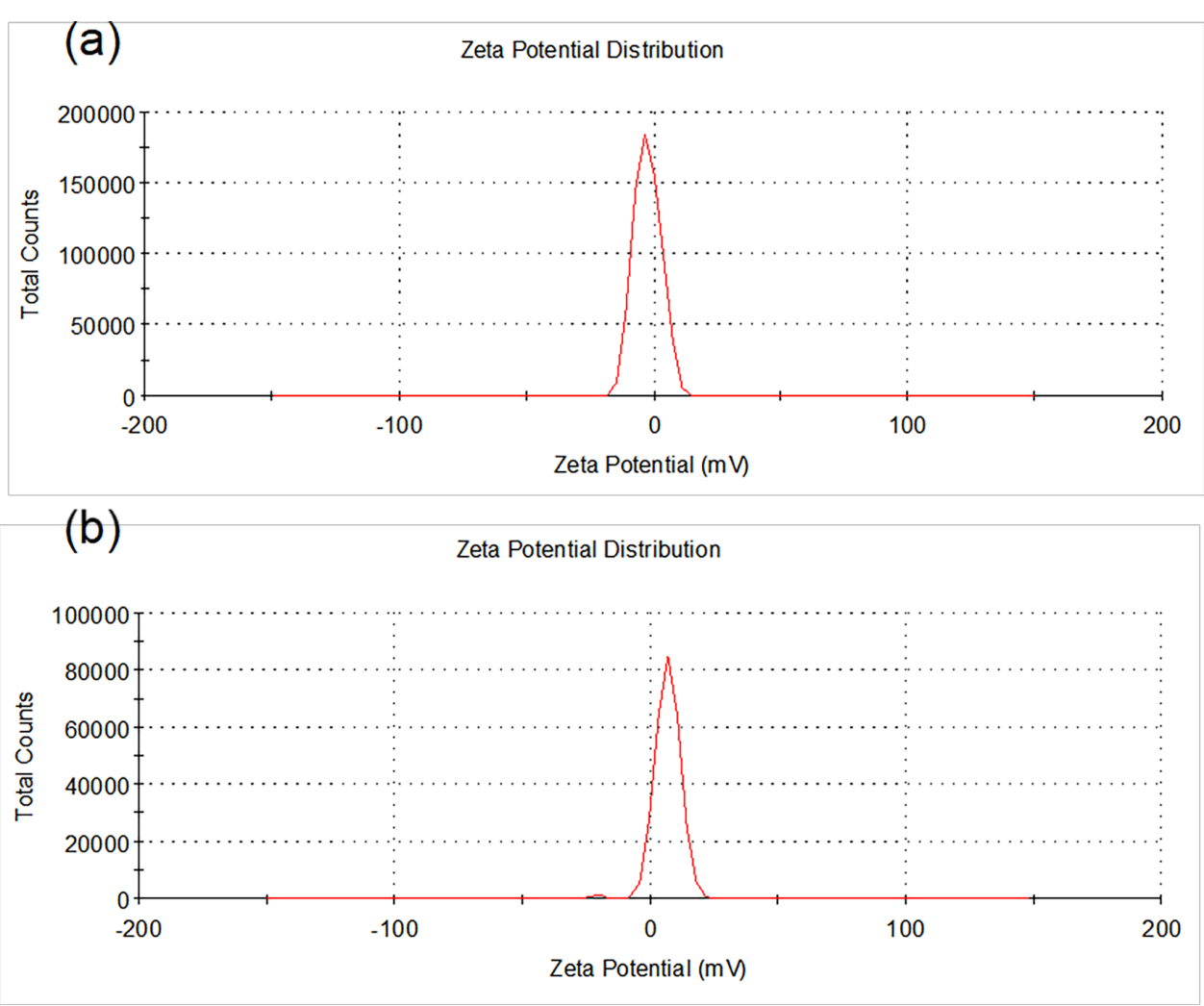


**Figure S2** The Zeta potentials of (a) Try-NaGdF_4_ NDs and (b) anti-CXCR4-NaGdF_4_ NDs.


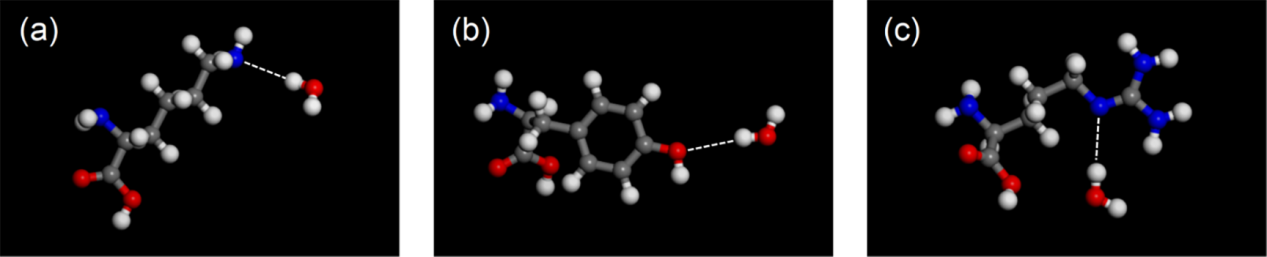


**Figure S3** The hydrogen bonds between (a) lysine, (b) tyrosine, (c) arginine and H_2_O molecules. The calculation of hydrogen bond energies between the side chain of polar amino acids (lysine, tyrosine and arginine) and H_2_O molecule was performed by the Quickstep module in CP2K. The spin-polarized Perdew-Burke-Ernzerhof (PBE) exchange-correlation functional combined with molecularly optimized double-zeta valence plus polarization (DZVP) basis set were adopted for electronic structure calculation. A 400 Ry energy was used for the Gaussian and Plane Wave (GPW) scheme of plane wave (PW) cutoff. Core electrons were modeled by Geodecker-Teter-Hutter (GTH) pseudo potentials with 5, 6, 1 and 4 valence electrons for N, O, H and C, respectively. Wavefunction analysis was calculated by Multiwfn package. The result shows that the hydrogen bond energies between the side chain of these amino acids (lysine, tyrosine and arginine) and H_2_O molecules were 21.16, 20.65, 22.14 kJ/mol, respectively.


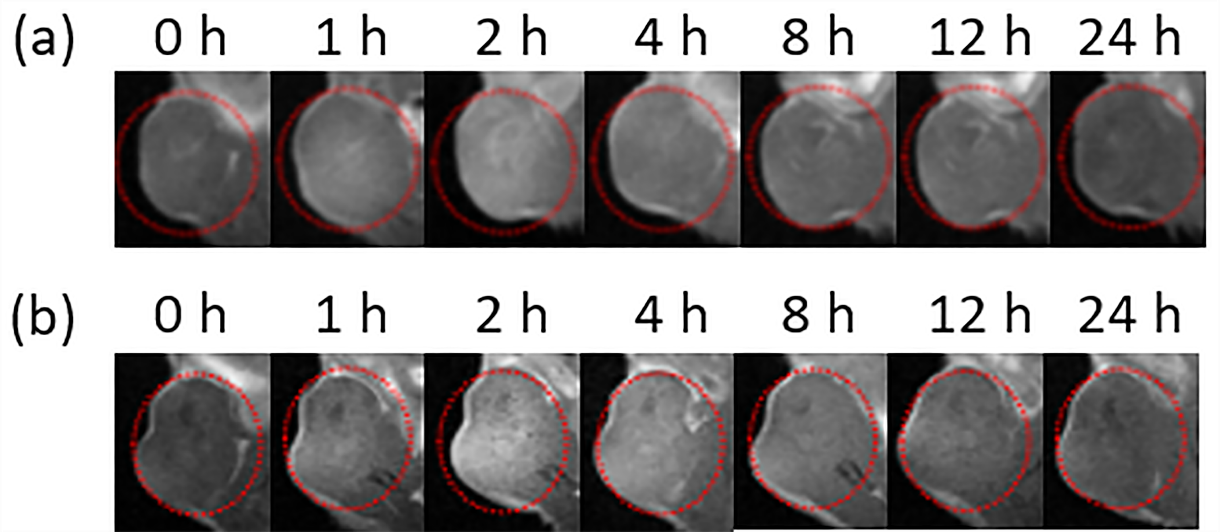


**Figure S4** In vivo MR images of tumor areas in BALB/c mice with MDA-MB-231 tumor after intravenous injection of (a) Try-NaGdF_4_ NDs and (b) anti-CXCR4-NaGdF_4_ NDs at pre-injection (0), 1, 2, 4, 8, 12 and 24 h post-injection, respectively. The injection dose is 10 mg [Gd] kg^−1^ body weight.


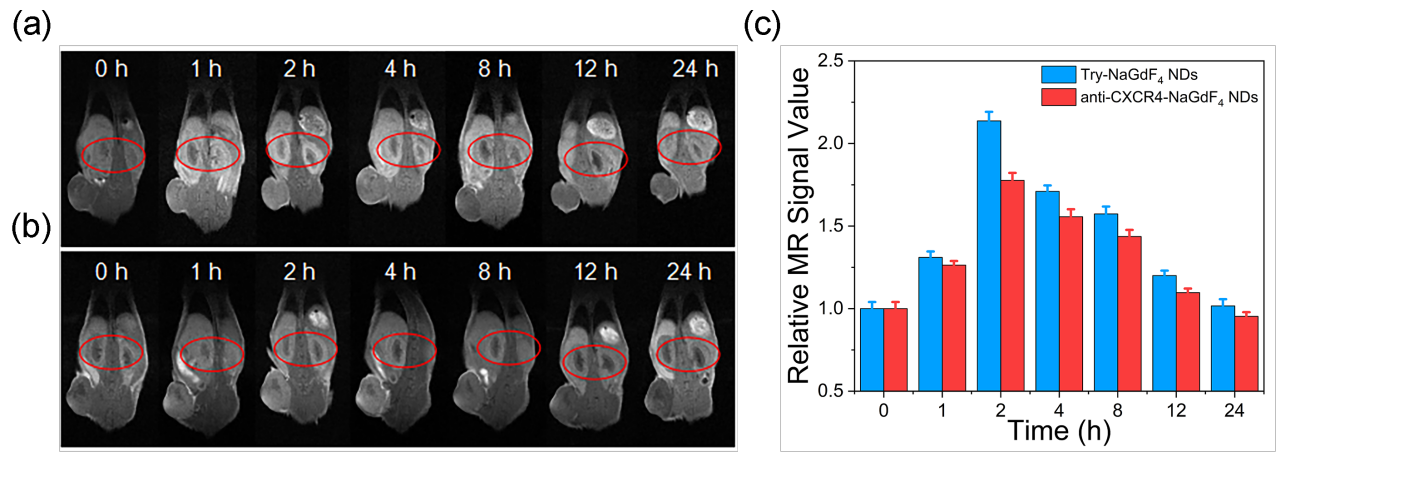


**Figure S5** In vivo MR images of BALB/c mice bearing the MDA-MB-231 tumor after intravenous injection of (a) Try-NaGdF_4_ NDs and (b) anti-CXCR4-NaGdF_4_ NDs at pre-injection (0), 1, 2, 4, 8, 12 and 24 h post-injection and (c) corresponding data analysis of the MR signal measurements of kidneys, respectively. The pre-injection (0) MR signal is defined as 1. The injection dose is 10 mg [Gd] kg^−1^ body weight.


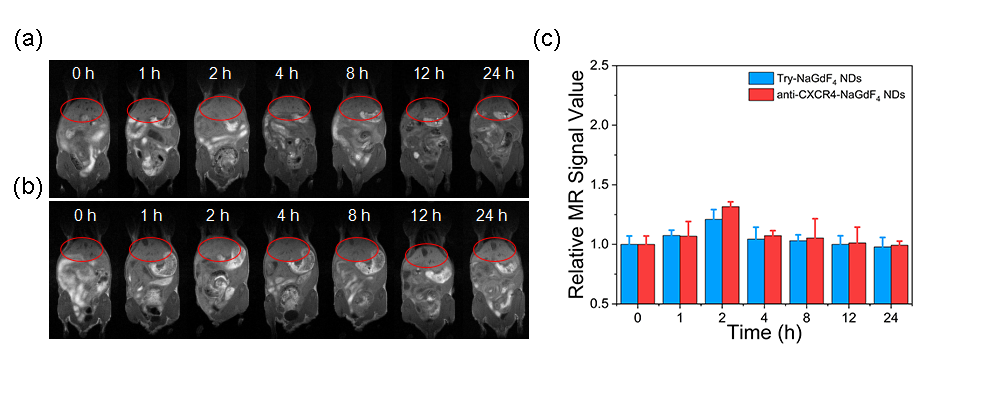


**Figure S6** In vivo MR images of BALB/c mice bearing the MDA-MB-231 tumor after intravenous injection of (a) Try-NaGdF_4_ NDs and (b) anti-CXCR4-NaGdF_4_ NDs at pre-injection (0), 1, 2, 4, 8, 12 and 24 h post-injection and (c) corresponding data analysis of the MR signal measurements of liver, respectively. The pre-injection (0) MR signal is defined as 1. The injection dose is 10 mg [Gd] kg^−1^ body weight.


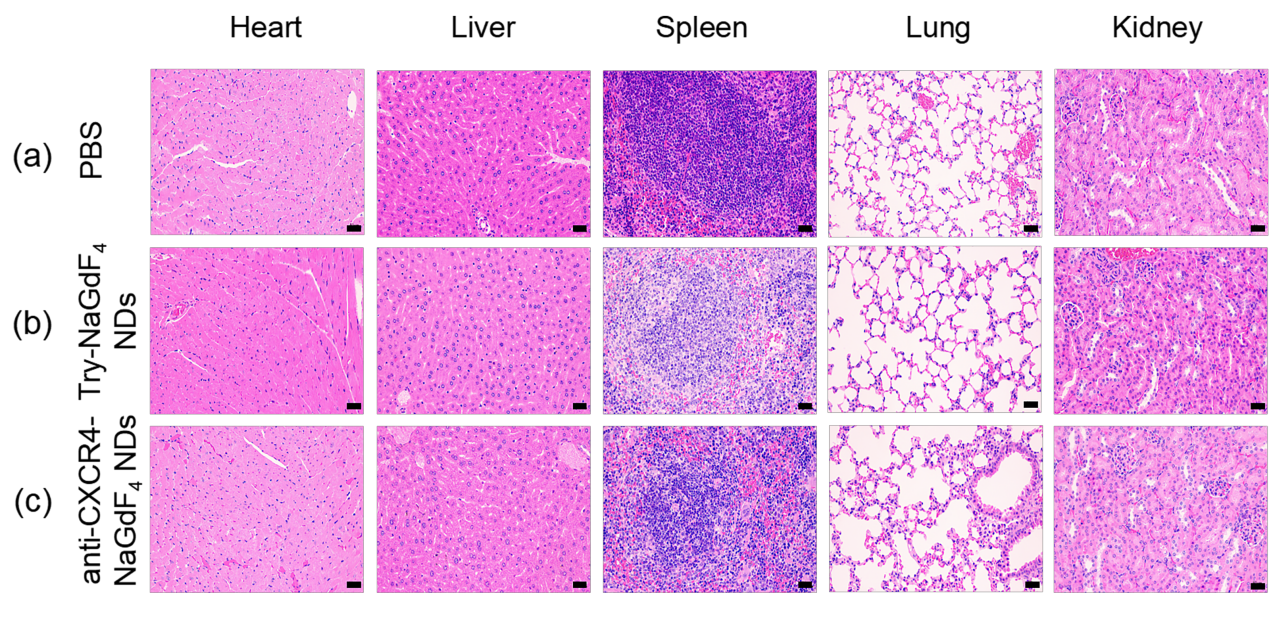


**Figure S7** H&E staining of major organs from healthy mice treated with (a) PBS, (b) Try-NaGdF_4_ NDs, and (c) anti-CXCR4-NaGdF_4_ NDs, respectively. The injection dose is 10 mg [Gd] kg^−1^ body weight. The scale bar is 50 μm.

**
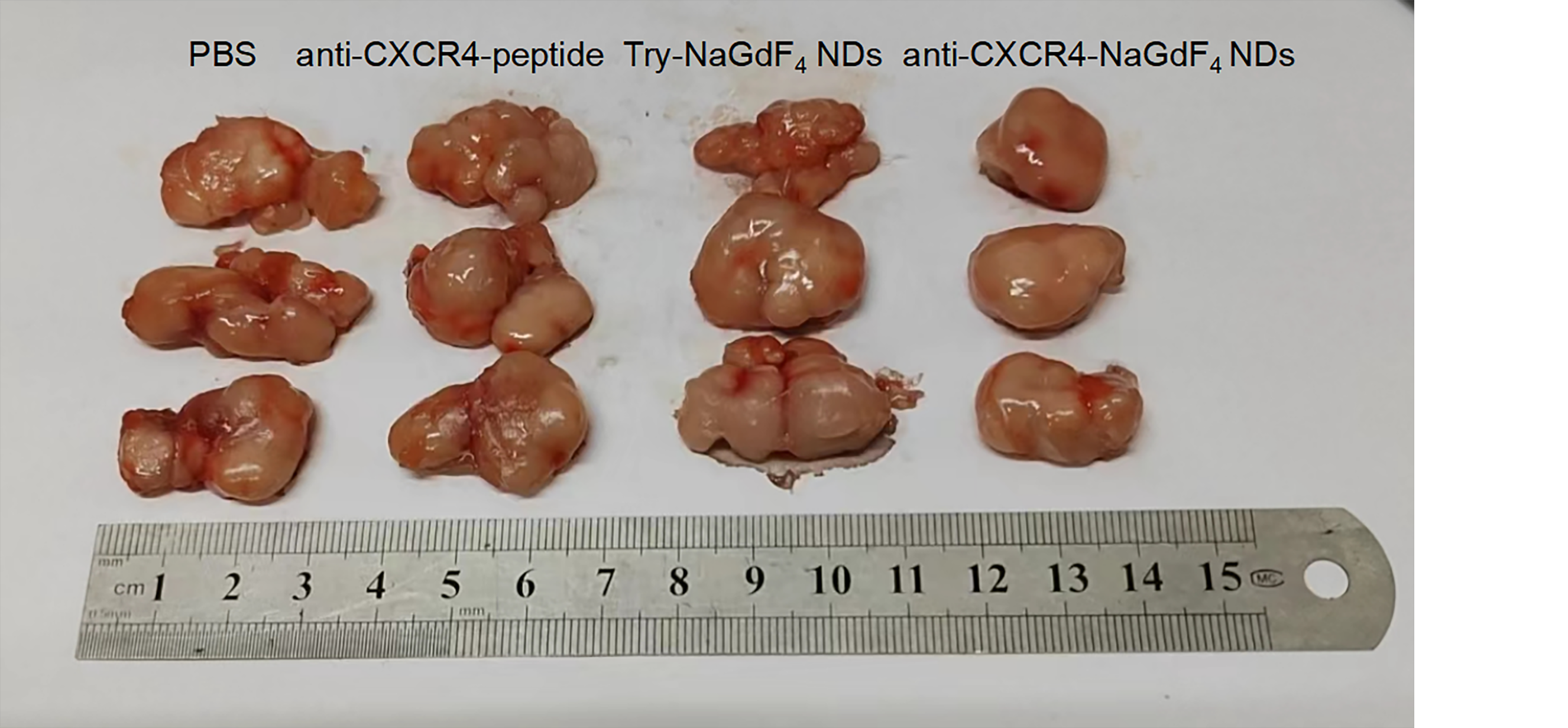
**

**Figure S8** The image of tumors collected from mice with different treatments.

**3 Additional References**

1. Johnson, N. J. J., Oakden, W., Stanisz, G. J., Scott Prosser, R. & van Veggel, F. C. J. M. Size-Tunable, Ultrasmall NaGdF_4_ Nanoparticles: Insights into Their T1 MRI Contrast Enhancement. *Chem. Mater.* **23,** 3714-3722 (2011).

2. Liu, F. Y., He, X. X., Zhang, J. P., Zhang, H. M. & Wang, Z. X. Employing Tryptone as a General Phase Transfer Agent to Produce Renal Clearable Nanodots for Bioimaging. *Small* **11,** 3676-3685 (2015).

3. Chen, H. D., Li, X. D., Liu, F. Y., Zhang, H. M. & Wang, Z. X. Renal Clearable Peptide Functionalized NaGdF_4_ Nanodots for High-Efficiency Tracking Orthotopic Colorectal Tumor in Mouse. *Mol. Pharm.* **14,** 3134-3141 (2017).
